# 1-Vinyl-1*H*-indole-3-carbaldehyde

**DOI:** 10.1107/S160053680801547X

**Published:** 2008-05-30

**Authors:** S. Selvanayagam, B. Sridhar, K. Ravikumar, S. Kathiravan, R. Raghunathan

**Affiliations:** a Department of Physics, Kalasalingam University, Krishnankoil 626 190, India; b Laboratory of X-ray Crystallography, Indian Institute of Chemical Technology, Hyderabad 500 007, India; c Department of Organic Chemistry, University of Madras, Guindy Campus, Chennai 600 025, India

## Abstract

In the title compound, C_11_H_9_NO, the C and O atoms of the attached carbaldehyde group deviate by just 0.052 (2) and 0.076 (1) Å, respectively, from the mean plane of the indole ring system. In addition to van der Waals forces, the mol­ecular packing is stabilized by C—H⋯O hydrogen bonds, which form a *C*(7) chain motif, and π–π inter­actions (centroid–centroid distance 3.637 Å) between the pyrrole and benzene rings of the indole ring system.

## Related literature

For related literature, see: Padwa *et al.* (1999 ▶); Mathiesen *et al.* (2005 ▶); Grinev *et al.* (1984 ▶); Gadaginamath & Patil (1999 ▶); Rodriguez *et al.* (1985 ▶); Karthick *et al.* (2005 ▶); Selvanayagam *et al.* (2005 ▶); Sonar *et al.* (2005 ▶). For bond-length data, see: Allen *et al.* (1987 ▶).
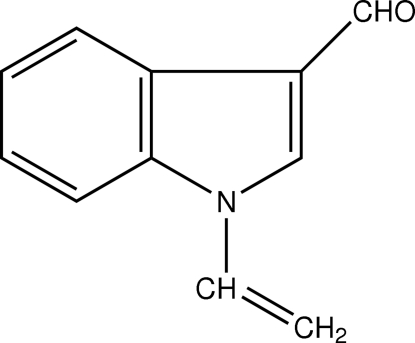


## Experimental

### 

#### Crystal data

C_11_H_9_NO
*M* *_r_* = 171.19
Monoclinic, 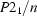

*a* = 8.3200 (5) Å
*b* = 8.1490 (5) Å
*c* = 13.1620 (7) Å
β = 99.952 (1)°
*V* = 878.95 (9) Å^3^
*Z* = 4
Mo *K*α radiation
μ = 0.08 mm^−1^
*T* = 293 (2) K
0.24 × 0.22 × 0.20 mm

#### Data collection

Bruker SMART APEX CCD area-detector diffractometer
Absorption correction: none
9730 measured reflections
2072 independent reflections
1823 reflections with *I* > 2σ(*I*)
*R*_int_ = 0.020

#### Refinement

*R*[*F* ^2^ > 2σ(*F* ^2^)] = 0.044
*wR*(*F* ^2^) = 0.127
*S* = 1.05
2072 reflections
118 parameters
H-atom parameters constrained
Δρ_max_ = 0.21 e Å^−3^
Δρ_min_ = −0.19 e Å^−3^

### 

Data collection: *SMART* (Bruker, 2001 ▶); cell refinement: *SAINT* (Bruker, 2001 ▶); data reduction: *SAINT*; program(s) used to solve structure: *SHELXS97* (Sheldrick, 2008 ▶); program(s) used to refine structure: *SHELXL97* (Sheldrick, 2008 ▶); molecular graphics: *ORTEP-3* (Farrugia, 1997 ▶) and *PLATON* (Spek, 2003 ▶); software used to prepare material for publication: *SHELXL97* and *PARST* (Nardelli, 1995 ▶).

## Supplementary Material

Crystal structure: contains datablocks I, global. DOI: 10.1107/S160053680801547X/bt2714sup1.cif

Structure factors: contains datablocks I. DOI: 10.1107/S160053680801547X/bt2714Isup2.hkl

Additional supplementary materials:  crystallographic information; 3D view; checkCIF report

## Figures and Tables

**Table 1:** Hydrogen-bond geometry (Å, °)

*D*—H⋯*A*	*D*—H	H⋯*A*	*D*⋯*A*	*D*—H⋯*A*
C9—H9⋯O1^i^	0.93	2.51	3.390 (2)	159

Symmetry code: (i) 
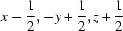
.

**Fig. 1.**
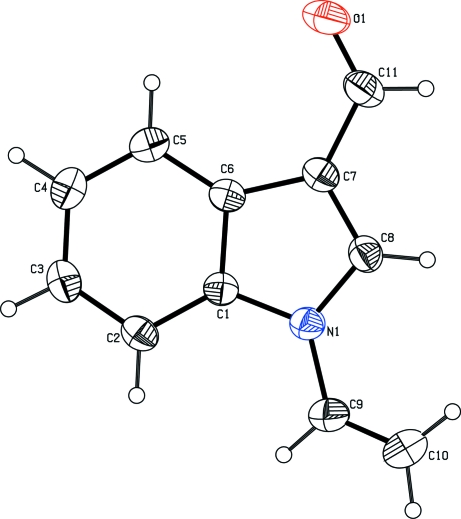
The structure and atom-numbering scheme for the title compound; displacement ellipsoids are drawn at the 30% probability level and H atoms are shown as small spheres of arbitrary radius.

**Fig. 2.**
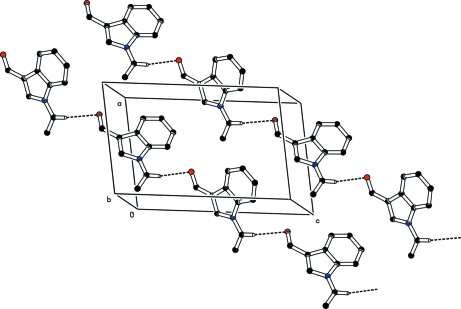
Molecular packing of the title compound viewed down the b axis; H-bonds are shown as dashed lines. For clarity, H atoms, not involved in hydrogen bonds, have been omitted.

## supplementary crystallographic information

### Comment

Indoles and their derivatives have been interest for many years, since large number of natural products contain indole systems and they are found in a number of pharmaceutical products, fragrances and dyes (Padwa *et al.*, 1999). Indole derivatives are identified as interfering with a G protein-independent signaling pathway of the CRTH2 receptor (Mathiesen *et al.*, 2005). These derivatives possess antidepressant (Grinev *et al.*, 1984), anti-microbial (Gadaginamath & Patil, 1999) and anti-inflammatory (Rodriguez *et al.*, 1985) activities. In view of its importance, we have undertaken the single-crystal X-ray diffraction study and report here its results.

The X-ray study confirmed the molecular structure and atomic connectivity for (I), as illustrated in Fig. 1. The geometry of the indole ring system is comparable to those reported for other indole derivatives (Karthick *et al.*, 2005; Selvanayagam *et al.*, 2005; Sonar *et al.*, 2005). The bond length of C9—C10 [1.284 (2) Å] confirms the double bond character (Allen *et al.*, 1987). The sum of the angles at N1 of the indole ring (360°) is in accordance with *sp*^2^ hybridization.

The indole ring is planar with a maximum deviation of 0.017 (1) Å for atom C8. The carbaldehyde group atoms C11 and O1 deviate 0.052 (2) and 0.076 (1) Å, respectively from the best plane of the indole ring.

In addition to the van der Waals forces, the molecular packing is stabilized by intermolecular C—H···O hydrogen bond (Table 2). Atom H9 of C9 forms a intermolecular hydrogen bond with oxygen atom O1 forming a C(7) chain motif of C—H···O hydrogen bond along the diagonal of *ac* plane (Fig. 2). In addition to this a weak π···π interaction between the pyrrole ring (N1/C1/C6—C8) at (*x*,*y*,*z*) and benzene ring (C1—C6) at (1 -*x*, -*y*), -*z*) stabilizes the molecular packing. The centroid-to-centroid distance is 3.637Å.

### Experimental

A mixture of *N*-vinylindole (0.05 mol) and DMF (0.15 mol) was stirred with POCl_3_ (32.3 ml). The reaction mixture was poured into ice water (300 ml) and stirred for 30minutes at less than 10° C. The precipitated solid was collected by filtration and washed well water (100 ml). In order to get the diffraction quality crystals, the compound was recrystallized from ethyl acetate.

### Refinement

The H atoms were positioned geometrically with C—H distances of 0.93 Å and were included in the refinement in the riding motion approximation with *U*_iso_= 1.2*U*_eq_(C).

### Crystal data

**Table:**

C_11_H_9_NO	*F*_000_ = 360
*M**_r_* = 171.19	*D*_x_ = 1.294 Mg m^−^^3^
Monoclinic, *P*2_1_/*n*	Mo *K*α radiation λ = 0.71073 Å
Hall symbol: -P 2yn	Cell parameters from 4554 reflections
*a* = 8.3200 (5) Å	θ = 2.1–23.6º
*b* = 8.1490 (5) Å	µ = 0.08 mm^−^^1^
*c* = 13.1620 (7) Å	*T* = 293 (2) K
β = 99.952 (1)º	Block, colourless
*V* = 878.95 (9) Å^3^	0.24 × 0.22 × 0.20 mm
*Z* = 4	

### Data collection

**Table:**

Bruker SMART APEX CCD area-detector diffractometer	1823 reflections with *I* > 2σ(*I*)
Radiation source: fine-focus sealed tube	*R*_int_ = 0.020
Monochromator: graphite	θ_max_ = 28.0º
*T* = 293(2) K	θ_min_ = 3.0º
ω scans	*h* = −10→10
Absorption correction: none	*k* = −10→10
9730 measured reflections	*l* = −17→16
2072 independent reflections	

### Refinement

**Table:**

Refinement on *F*^2^	Secondary atom site location: difference Fourier map
Least-squares matrix: full	Hydrogen site location: inferred from neighbouring sites
*R*[*F*^2^ > 2σ(*F*^2^)] = 0.044	H-atom parameters constrained
*wR*(*F*^2^) = 0.127	*w* = 1/[σ^2^(*F*_o_^2^) + (0.0665*P*)^2^ + 0.1375*P*] where *P* = (*F*_o_^2^ + 2*F*_c_^2^)/3
*S* = 1.05	(Δ/σ)_max_ < 0.001
2072 reflections	Δρ_max_ = 0.21 e Å^−^^3^
118 parameters	Δρ_min_ = −0.19 e Å^−^^3^
Primary atom site location: structure-invariant direct methods	Extinction correction: none

### Special details

**Table:**

Geometry. All e.s.d.'s (except the e.s.d. in the dihedral angle between two l.s. planes) are estimated using the full covariance matrix. The cell e.s.d.'s are taken into account individually in the estimation of e.s.d.'s in distances, angles and torsion angles; correlations between e.s.d.'s in cell parameters are only used when they are defined by crystal symmetry. An approximate (isotropic) treatment of cell e.s.d.'s is used for estimating e.s.d.'s involving l.s. planes.
Refinement. Refinement of *F*^2^ against ALL reflections. The weighted *R*-factor *wR* and goodness of fit *S* are based on *F*^2^, conventional *R*-factors *R* are based on *F*, with *F* set to zero for negative *F*^2^. The threshold expression of *F*^2^ > σ(*F*^2^) is used only for calculating *R*-factors(gt) *etc*. and is not relevant to the choice of reflections for refinement. *R*-factors based on *F*^2^ are statistically about twice as large as those based on *F*, and *R*- factors based on ALL data will be even larger.

### Fractional atomic coordinates and isotropic or equivalent isotropic displacement parameters (Å^2^)

**Table:**

	*x*	*y*	*z*	*U*_iso_*/*U*_eq_	
O1	0.81283 (15)	0.20464 (18)	−0.13362 (9)	0.0826 (4)	
N1	0.41791 (12)	0.24005 (13)	0.07004 (7)	0.0469 (3)	
C1	0.54641 (14)	0.14623 (13)	0.12226 (8)	0.0424 (3)	
C2	0.56155 (16)	0.06833 (16)	0.21713 (9)	0.0505 (3)	
H2	0.4800	0.0748	0.2572	0.061*	
C3	0.70276 (18)	−0.01899 (17)	0.24918 (10)	0.0581 (3)	
H3	0.7166	−0.0736	0.3121	0.070*	
C4	0.82544 (17)	−0.02764 (17)	0.18966 (11)	0.0594 (4)	
H4	0.9197	−0.0870	0.2139	0.071*	
C5	0.80997 (15)	0.04980 (16)	0.09564 (10)	0.0527 (3)	
H5	0.8925	0.0434	0.0563	0.063*	
C6	0.66781 (14)	0.13815 (13)	0.06061 (8)	0.0431 (3)	
C7	0.60762 (15)	0.23003 (15)	−0.03148 (9)	0.0480 (3)	
C8	0.45684 (16)	0.28725 (16)	−0.02188 (9)	0.0503 (3)	
H8	0.3901	0.3497	−0.0712	0.060*	
C9	0.27187 (17)	0.27412 (19)	0.10596 (12)	0.0615 (4)	
H9	0.2701	0.2521	0.1751	0.074*	
C10	0.14066 (19)	0.3327 (2)	0.05257 (15)	0.0779 (5)	
H10A	0.1365	0.3567	−0.0169	0.094*	
H10B	0.0498	0.3511	0.0834	0.094*	
C11	0.68162 (19)	0.2565 (2)	−0.12120 (11)	0.0621 (4)	
H11	0.6233	0.3191	−0.1742	0.075*	

### Atomic displacement parameters (Å^2^)

**Table:**

	*U*^11^	*U*^22^	*U*^33^	*U*^12^	*U*^13^	*U*^23^
O1	0.0720 (7)	0.1185 (10)	0.0649 (7)	0.0021 (6)	0.0336 (6)	0.0075 (6)
N1	0.0474 (5)	0.0518 (6)	0.0428 (5)	0.0032 (4)	0.0110 (4)	−0.0026 (4)
C1	0.0457 (6)	0.0418 (5)	0.0396 (5)	−0.0022 (4)	0.0075 (4)	−0.0069 (4)
C2	0.0595 (7)	0.0521 (7)	0.0417 (6)	−0.0035 (5)	0.0136 (5)	−0.0023 (5)
C3	0.0707 (8)	0.0549 (7)	0.0464 (6)	0.0007 (6)	0.0035 (6)	0.0051 (5)
C4	0.0562 (7)	0.0544 (7)	0.0641 (8)	0.0080 (6)	0.0011 (6)	0.0007 (6)
C5	0.0475 (6)	0.0527 (7)	0.0591 (7)	0.0000 (5)	0.0129 (5)	−0.0070 (5)
C6	0.0472 (6)	0.0418 (6)	0.0411 (5)	−0.0053 (4)	0.0102 (4)	−0.0072 (4)
C7	0.0543 (7)	0.0486 (6)	0.0425 (6)	−0.0046 (5)	0.0124 (5)	−0.0023 (5)
C8	0.0566 (7)	0.0513 (7)	0.0427 (6)	0.0017 (5)	0.0079 (5)	0.0021 (5)
C9	0.0564 (8)	0.0736 (9)	0.0586 (8)	0.0089 (6)	0.0209 (6)	−0.0013 (6)
C10	0.0580 (9)	0.0892 (12)	0.0894 (12)	0.0170 (8)	0.0205 (8)	0.0036 (9)
C11	0.0670 (8)	0.0743 (9)	0.0481 (7)	−0.0059 (7)	0.0185 (6)	0.0041 (6)

### Geometric parameters (Å, °)

**Table:**

O1—C11	1.2079 (19)	C5—C6	1.3933 (17)
N1—C8	1.3610 (16)	C5—H5	0.9300
N1—C1	1.3949 (15)	C6—C7	1.4390 (17)
N1—C9	1.4055 (16)	C7—C8	1.3645 (18)
C1—C2	1.3869 (16)	C7—C11	1.4393 (18)
C1—C6	1.4023 (16)	C8—H8	0.9300
C2—C3	1.3760 (19)	C9—C10	1.284 (2)
C2—H2	0.9300	C9—H9	0.9300
C3—C4	1.392 (2)	C10—H10A	0.9300
C3—H3	0.9300	C10—H10B	0.9300
C4—C5	1.3751 (19)	C11—H11	0.9300
C4—H4	0.9300		
			
C8—N1—C1	108.24 (10)	C5—C6—C1	119.20 (11)
C8—N1—C9	126.55 (12)	C5—C6—C7	134.39 (11)
C1—N1—C9	125.17 (11)	C1—C6—C7	106.40 (10)
C2—C1—N1	129.57 (11)	C8—C7—C6	106.95 (10)
C2—C1—C6	122.48 (11)	C8—C7—C11	123.72 (13)
N1—C1—C6	107.94 (10)	C6—C7—C11	129.29 (12)
C3—C2—C1	116.91 (12)	N1—C8—C7	110.46 (11)
C3—C2—H2	121.5	N1—C8—H8	124.8
C1—C2—H2	121.5	C7—C8—H8	124.8
C2—C3—C4	121.60 (12)	C10—C9—N1	126.30 (15)
C2—C3—H3	119.2	C10—C9—H9	116.8
C4—C3—H3	119.2	N1—C9—H9	116.8
C5—C4—C3	121.31 (12)	C9—C10—H10A	120.0
C5—C4—H4	119.3	C9—C10—H10B	120.0
C3—C4—H4	119.3	H10A—C10—H10B	120.0
C4—C5—C6	118.49 (12)	O1—C11—C7	125.68 (15)
C4—C5—H5	120.8	O1—C11—H11	117.2
C6—C5—H5	120.8	C7—C11—H11	117.2
			
C8—N1—C1—C2	−178.34 (12)	N1—C1—C6—C7	−0.32 (12)
C9—N1—C1—C2	−0.3 (2)	C5—C6—C7—C8	178.96 (13)
C8—N1—C1—C6	0.77 (13)	C1—C6—C7—C8	−0.24 (13)
C9—N1—C1—C6	178.86 (12)	C5—C6—C7—C11	1.3 (2)
N1—C1—C2—C3	178.96 (11)	C1—C6—C7—C11	−177.94 (13)
C6—C1—C2—C3	−0.04 (18)	C1—N1—C8—C7	−0.95 (14)
C1—C2—C3—C4	0.56 (19)	C9—N1—C8—C7	−179.00 (12)
C2—C3—C4—C5	−0.6 (2)	C6—C7—C8—N1	0.73 (14)
C3—C4—C5—C6	0.0 (2)	C11—C7—C8—N1	178.60 (12)
C4—C5—C6—C1	0.47 (17)	C8—N1—C9—C10	11.7 (3)
C4—C5—C6—C7	−178.65 (12)	C1—N1—C9—C10	−166.00 (16)
C2—C1—C6—C5	−0.47 (17)	C8—C7—C11—O1	−178.09 (15)
N1—C1—C6—C5	−179.66 (10)	C6—C7—C11—O1	−0.7 (3)
C2—C1—C6—C7	178.87 (10)		

### Hydrogen-bond geometry (Å, °)

**Table:**

*D*—H···*A*	*D*—H	H···*A*	*D*···*A*	*D*—H···*A*
C9—H9···O1^i^	0.93	2.51	3.390 (2)	159

Symmetry codes: (i) *x*−1/2, −*y*+1/2, *z*+1/2.

## References

[bb1] Allen, F. H., Kennard, O., Watson, D. G., Brammer, L., Orpen, A. G. & Taylor, R. (1987). *J. Chem. Soc. Perkin Trans. 2*, pp. S1–19.

[bb2] Bruker (2001). *SAINT* and *SMART* Bruker AXS Inc., Madison, Wisconsin, USA.

[bb3] Farrugia, L. J. (1997). *J. Appl. Cryst.* **30**, 565.

[bb4] Gadaginamath, G. S. & Patil, S. A. (1999). *Indian J. Chem. B*, **38**, 1070–1074.

[bb5] Grinev, A. N., Shevdov, V. L., Krichevskii, E. S., Romanova, O. B., Altukkhova, L. B., Kurilo, G. N., Andreeva, N. I., Golovina, S. M. & Mashkovskii, M. D. (1984). *Khim. Farm. Zh.* **18**, 159–163.

[bb6] Karthick, S., Selvanayagam, S., Velmurugan, D., Ravikumar, K., Arumugam, N. & Raghunathan, R. (2005). *Acta Cryst.* E**61**, o1780–o1782.

[bb7] Mathiesen, J. M., Ulven, T., Martini, L., Gerlach, L. O., Heinemann, A. & Kostenis, E. (2005). *Mol. Pharmacol.* **68**, 393–402. 10.1124/mol.104.010520 15870392

[bb8] Nardelli, M. (1995). *J. Appl. Cryst.* **28**, 659.

[bb9] Padwa, A., Brodney, M. A., Liu, B., Satake, K. & Wu, T. (1999). *J. Org. Chem.* **64**, 3595–3607. 10.1021/jo982453g 11674487

[bb10] Rodriguez, W. G., Temprano, F., Esteban-Calderon, C., Martinez-Ripoll, M. & Garcia-Blanco, S. (1985). *Tetrahedron*, **41**, 3813–3823.

[bb11] Selvanayagam, S., Chandak, M. S., Velmurugan, D., Ravikumar, K. & Raghunathan, R. (2005). *Acta Cryst.* E**61**, o3122–o3123.

[bb12] Sheldrick, G. M. (2008). *Acta Cryst.* A**64**, 112–122. 10.1107/S0108767307043930 18156677

[bb13] Sonar, V. N., Parkin, S. & Crooks, P. A. (2005). *Acta Cryst.* C**61**, o78–o80. 10.1107/S0108270104032925 15695916

[bb14] Spek, A. L. (2003). *J. Appl. Cryst.* **36**, 7–13.

